# Supplementary material for: Discharge pharmacotherapy for Type 2 diabetic inpatients at two hospitals of different tiers in Zhejiang Province, China
Source: PLoS One. 2020 Apr 8;15(4):e0230123. doi: 10.1371/journal.pone.0230123 (PMC7141672; doi:10.1371/journal.pone.0230123)
Supplement: Attachment — Submitted filename: Response to Reviewers.docx [file pone.0230123.s001.docx]

Dear Editor

We thank the reviewers for their meticulous work and generous comments on the manuscript and we have revised the manuscript to address their concerns point by point.

Journal requirements are also carefully checked and followed through. We hope it will meet the publishing criteria now. Look forward to hearing from you soon.

Happy New Year. Best regards from

Xiaofang Yu

2020-01-13

Reviewer #1:

***This article assessed the service use among Type 2 diabetes in two different levels of hospitals in Ningbo, Eastern China. While understanding the service use is important, quite many articles have already published in this field. For the selected hospitals in the article, it is also difficult to argue the disparity of service used among patients, since the tertiary may treat more patients with severe conditions or complications. In addition, the impact of the hospital alliance is uncertain*.**

***The research design is valid but the English writing needs improvement.***

Truly so. Inpatient management of T2DM has been a popular field of discussion and has arrived at some consensus. Yet discharge arrangements in T2DM inpatients remain overlooked. We consider our focus on discharge pharmacology as one highlight since lack of consensus on this is a common deficiency in nowadays clinical guidelines. Chinese guideline of diabetes management neglects the discharge transition in its chapter of in-hospital diabetes care [1 ] . Though American Diabetes Association (ADA) guideline has included the discharge process in the part of in-hospital diabetes care, it provides no approach for transition of pharmacologic management from inpatient to outpatient care [2].We figure there would be great disparities in this part of diabetes management. With this in mind, we choose uncomplicated T2DM in this analysis to take a look at the real-world diabetes management in one health alliance. Taking these facts into consideration, we believe the analysis has its merits and provides one good perspective.

It’s true that LHLH as a tertiary hospital treats more patients with severe conditions. As a fact, in the construction of health alliance, better dissemination of standardized management of uncomplicated T2DM and orderly dual-referral systems for complicated T2DM are two cornerstones. Our analysis here is of one perspective to the current situation. We focus on T2DM uncomplicated with comorbidities and organ dysfunctions that may justify further referral (Another article discussed the situation of the dual-referral system in Ningbo in the process, not published yet.) Thanks to your reminding we have elaborated on the health alliance in the INTRODUCTION to better clarify the background.

We have also checked the language to avoid typographical or grammatical errors.

***Abstract***

***It is not clear to me, are authors want to detect the deficiencies in discharge management by comparison between the two hospitals?***

No, we didn’t mean to detect deficiencies in discharge management by comparison between the two hospitals. I realized that it’s my expression that caused confusion and revision has been made. Generally speaking, insufficient discharge management is common partly because the current Chinese guidelines give no specific comments on it. Our study focused on discharge pharmacotherapy as a perspective to the problem. So it’s not that the deficiencies in discharge management are detected by comparison between the two hospitals. Rather, it was studied in two hospitals.

***Is this analysis performed on patients’ medical records? How to identify eligible patients? what time is this analysis conducted?***

Yes. This analysis is performed on patients’ medical records obtained from hospital information system (HIS) of two hospitals. Data collectors search HIS for patients with the principal diagnosis being type 2 diabetes, or, the ICD-10 coding being E11. Then the patients’ entire records were scrutinized and those with no doubt in diabetes classification and with no conditions in exclusion criteria were considered eligible and included for further analysis. Records were accessed and data collected during May and August 2019 before data analyzing was carried out by another analyst. We’ve now supplemented the METHODS with the necessary information to avoid confusion based on your reasonable concerns.

***Introduction
Is there any published clinical guideline for diabetes in China? And its implementation?
A proper literature review need to be conducted and describe the additional value of this article to published work.***
 Yes. China has published clinical guideline for diabetes management and kept updating it with ever-emerging new evidences. So far Chinese Diabetes Society (CDS) has published five versions of guidelines for diabetes management with the latest version released in 2018 [1]. CDS also takes great endeavor to promote its implementation and has achieved big progression so far. Chinese diverse demographics and imbalanced distribution of resources are major obstacles to this progression. The policy of hierarchical diabetes management is one of major measures taken in the process. Our study inspects current situation in diabetes management at two hospitals from one medical alliance (which can be considered as a basic functional unit of the policy). The reason why we choose discharge pharmacotherapy as an entry point is that deemphasizing of discharge management remains to be a common problem. There are still scare studies focusing on discharge medication transition in the literature. These were considerations make us believe our work has its merits over published work. We’ve briefly elaborated on this in INTRODUCTION but without a thorough review of it, but we could if you would like.

***Methods:
Patients were included from the two hospitals from different year. Will this influence results?
Any calculation to get the sample size?***

Yes it may influence the results and we honestly expected this may arouse concerns. We originally set the starting time in Jan 2017 since it’s time that SMH developed an extensive trusteeship with LHLH and achieved considerable development. The reason why we choose different year (Jan 2018 rather than Jan 2017 as at SMH) at LHLH was actually simple but awkward. There was an update of hospital information system at LHLH resulted in some deficits in the interface of former medical electronic records with the laboratory information system. Though with all laboratory data still retrievable, it requires extra training of data collectors. Since we estimated that a sample size of less than 100 (65) patients at each hospital was necessary｛with insulin treatment assuming 60% [3 ] ( 50% at LHLH, 70% at SMH according to the rough estimation of our data ) , OADs-only treatment assuming 40%, to reach a 90% chance to detect the difference, PASS v2019.0.2｝, we settled for one year’s data at LHLH with a little more than double of patients eligible compared to SMH. We didn’t explain this in the manuscript, but we could supplement it as another limitation if it’s acceptable.

***If hospital was input into the regression model as dummy variable, what will happen? Will “hospital” have significance in treatment when control other variables?***
Thank you so much for the enlightening question. Based on your suggestion we adjusted our statistical strategy. We applied generalized linear models (GLM) with mixed effects including ‘hospital’ to study influences of discharge pharmacology, which verified ‘hospital’ as a significant impact on treatment (OR: 0.352, 95%CI [0.213, 0.570]). We then extended GLM to generalized linear mixed models (GLMM) by incorporating ‘hospital’ as the random effect and other variables as fixed effects to account for differences between insulin treatment and OADs-only treatment. Results are presented in Table 5 in the manuscript (*Line247-248*).

***Reviewer #2***

*The authors conducted a retrospective cross-sectional study of the discharge pharmacotherapy for type 2 diabetes inpatients in their two hospitals of different tiers. Data is relevant for publication, since there doesn’t exist similar information, especially in a new type of medical referral system named “Regional Longitudinal Health Alliance” in China. The knowledge of the pharmacotherapy in this particular population and setting could be used by Chinese experts in developing, evaluating implementing interventions for optimizing diabetes managements. In general, I appreciated this manuscript. Although it is conducted in a scientifically meaningful way, there are still some limitations which should be clarified.* ***1. Line90-93: Please provide more information on the “Regional Longitudinal Health Alliance”. For example, how the hospitals of different tiers in the Health Alliance interact with each other? Is this just a referral system? How does this Health Alliance improve the management of T2DM?***

Revision has been made. Thank you for the reminding and we’ve realized it’s essential to elaborate more in detail on the health alliance (as in *Line 76-92* in the manuscript). It’s not just a referral system. It could be considered as a basic functional unit in executing hierarchical diabetes management, of which orderly dual-referral systems for complicated T2DM and better dissemination of standardized management of uncomplicated T2DM are two cornerstones. The construction of health alliances is a multi-sectoral collaboration involving administrative, financial, educational, as well as clinical affairs. An effective dual-referral flow of complicated T2DM makes sure that complicated T2DM, if in need of advanced health technology for diagnosis, evaluation or treatment, could be timely referred from subordinate institutions to tertiary hospitals; and once stabilized, could get back to the subordinate institutions with treatment plans and acquire a smooth transition from acute/critical treatment to standardized chronic disease management. Improvement of disease management in primary and secondary institutions are achieved with multiple measures via various channels such as continuing education classes, short term training programs, periodical an on-demand consultation and so on. As the progression Ningbo witnesses, the development of health alliances is still in process. But in the long run will play a greater role in improving Chinese diabetes management.

***2. Line97-99: I recommend to delete this sentence. Although patients can skip the referral system and choose any hospitals now, I think it will take time to implement as new rules.***

Agreed. It’s a process to develop an efficient dual-referral system and to make appropriate regulations to assure its enforcement. We provided information about the current situation to better explain the reason why we chose SMH as the representative secondary hospital for now. We’ve edited in our manuscript to avoid confusion.  ***3. Line91: Please provide more information on tertiary hospital, secondary hospital and community healthcare centers.***

Revised (as in *Line 78-83*). Chinese accreditation system for hospital classified hospitals into three levels with each level further classified as class A/B/C. The synthetic strength of hospitals including hardware and software facilities, technology, administrative management and personnel proficiency, etc. is major assessment criteria. Tertiary hospitals accredited as Class 3A level are top providers followed with Class 3B, Class3C and then secondary hospitals accredited as Class 2A/2B/2C and so on. We have supplemented the INTRODUCTION with this information as you suggested.  ***4. Line125: Please clarify the definition of “clinically relevant hepatic disease”.***

Clinically relevant hepatic disease here is defined as ‘plasma transaminase ≥ 2.5 times the upper limit, whatever the causes are; or, cirrhosis, with or without biochemical abnormalities’. We’ve added the definition to the manuscript (*Line 135-137*).  ***5. Line128: Please readjust the serial number of exclusion criteria, which is a small but obvious mistake.***

Corrected and we apologize for the negligence. ***6. Line 128-134: I recommend to move these sentences to the discussion, as they are not part of the methods.***

Advice taken. This part was discussed at the beginning of discussion (*Line 251-258*). ***7. Line 140: Please clarify the definition of “cerebral vascular disease”. Ischemic stroke or hemorrhagic stroke?***

Cerebral vascular disease here includes ischemic as well as hemorrhagic stroke and is established on recorded history without extra verification. To avoid confusion we’ve unified the description as cerebral vascular diseases and necessary information provided in the METHOD (*Line 148-151*). ***8. Line 138: In table 1, you mentioned the variable “newly diagnosed diabetes”, but it was not mentioned in the methods, please add it and clarify the definition of “newly diagnosed diabetes”.***

Edited. Newly diagnosed diabetes in this analysis include patients that were diagnosed as diabetes for the first time, no matter whether they provided a long history of osmotic symptoms or not. Patients previously knew themselves as ‘diabetes’ or ‘hyperglycemia’ were not included as ‘newly diagnosed’, be them treated or not. We’ve added it to the METHOD in *Line 130-133* as well as in Table1.

***9. Line155-156: Has informed consent been obtained by participants? Probably not, as there was no access to individual data by the first author/corresponding author. Please indicate so in methods and in Line155.***

As you understand, informed consent forms were waivered for this retrospective and anonymized analysis. Information was provided in the METHOD along with the ethics statement in *Line118-125*.  ***10. Line140-141: Please provide more information of the diagnostic process of the micro- and macro-vascular complications.***

Revised. We’ve included it in the METHODS (*Line 145-157*). In this analysis, macro-vascular diseases were represented by peripheral artery diseases (carotid artery atherosclerosis and/or lower limb artery atherosclerosis), coronary heart disease and cerebral vascular diseases. The diagnosis of peripheral artery diseases at LHLH was made by the routine screening with doppler ultrasonography for all T2DM inpatients while at SMH peripheral artery diseases were not routinely screened. The latter two (coronary heart disease and cerebral vascular disease) conditions were mostly based on past history diagnosis at both hospitals. But at LHLH, patients with suspected symptoms were getting further examination like coronary CT or brain MRI to get diagnosed. Disparities exist in the establishment of micro-vascular diseases (represented as diabetic kidney diseases, DKD and diabetic retinopathy, DR) as well. At LHLH, DKD is diagnosed with urinary microalbumin creatinine ratio, UACR ＞30mg/g and/or estimated glomerular filtration rate, eGFR＜60.ml. min^-1^. (1.73m^2^)^-1^ and DR diagnosed with the international clinical grading standard for diabetic retinopathy. But at SMH, UACR has not been routinely carried out and DKD was diagnosed mostly based on serum creatinine and DR was also based on past history diagnosis. We admitted this in the LIMITATION as the diagnosis of vascular complications not so strict and convincing.  ***11. Line 153-154: Please provide more information of the regression analysis. For example, which independent variables were selected in the regression analysis?***

Edited. Taken your opinion of Table5 together into consideration, we’ve adjusted our statistical strategy and presented the new results in Table5 with explanatory information (*Line240-248*). We adopted the generalized linear mixed models with ‘hospital’ as the random effect and all other variables as fixed effect to study differences between insulin treatment and OADs-only treatment and inferred long duration of diabetes, high HbA1c concentration and vascular complications as predictor of insulin treatment on discharge at two hospitals.

***12. Line 164-165: Please recheck the data listed in this sentence and in table 1.***

There were 96 (24.6%) patients at LHLH and 84 (51.2%) patients at SMH were not on any antidibetes treatment on admission, among whom 74 (18%) at LHLH、54(32.9%) patients at SMH were newly diagnosed, leaving 22 (5.6%) patients at LHLH and 30 (18.3%) patients at SMH with known diabetes untreated before admission. That’s why we describe the result as ‘about 20% and 5% of the known T2DM at SMH and at LHLH, respectively, were untreated’(*Line183-184*). But we did omit ‘at LHLH’ in the manuscript. It’s a careless omission and we apologize for this.

Checked and corrected. ***13. Line 173 table 1: Please recheck the data in table 1. There is 88(53.1) in the row of Illiterate and the column of SMH, 74(91.4) in the row of Insurance-covered and the column of SMH. You should double-check these data whether they are right or not.***

Checked and corrected. Again, it’s caused by negligence. We feel really sorry for it and thank you truly for your meticulous proofing.  ***14. Line 173 table 1: In the row of BMI in table 1, please maintain 2 digits at most after decimal point. Same as in table 3 and table 4.***

Checked and corrected.  ***15. Line 173 table 1: Patients in both hospitals have a long duration (>9 years) and poor glycemic control (HbA1c>9.5%), but the incidence of diabetic micro-vascular complications is relevant low, especially in SMH. Please discuss.***

Totally agree. As we explained in your aforementioned concerns that establishment of vascular complications is one major limitation to our data, especially in patients from SMH. Take diagnosis of nephropathy as an example, at SMH, the diagnosis was mostly made on abnormal serum creatinine, which could miss more than half of patients with CKD compared to eGFR (6% vs 15.8%) as one study on kidney function in Chinese population found [4], let alone the fact that albuminuria was also underdiagnosed. So is the case of diabetic retinopathy. The low incidence actually shows one major deficiency in diabetes management at lower institutions like SMH that chronic complications don’t get diagnosed till advanced stages and awaits improvement in the future.

As to data from LHLH, it’s true that incidence of DKD and DR were both unexpectedly low with routinely screening. Even with the fact that results of studies on the DKD prevalence in Chinese population are with variance as great as 15%~60% [5, 6 ], it’s more commonly seen as 20%~40% [7].Except for possible influences like demographics and socioeconomic factors (familial predisposition, diet, smoking, etc.) in the target patients, we try to discuss from several aspects. 1. risk factors : Just as you pointed out, hyperglycemia and diabetes duration are established risk factors of microvascular diseases in T2DM, though the highest incidence rates are seen between 10 to 20 years after T2DM onset [7, 8 ]. Our patients did seem to be alleviated from some other risk factors like advanced age (＞ 65years), obesity, insulin resistance and kidney dysfunction [8],though. Pity that we didn’t include other possible relevant factors like lipids and uric acid that may also affect the risk of microvascular disease. 2. DKD phenotypes: the possibility of non-proteinuric DKD has been increasingly recognized in T2DM [9], this phenotype of DKD might differ in risk factors and susceptible population [10]. Should our patients tend to develop kidney function impairment over proteinuria, the exclusion of kidney insufficiency could impact the results to a greater extent. As a fact, non-proteinuric DKD was more frequently observed in the absence of diabetic retinopathy, which may also contribute to the even lower incidence of DR in this population. 3. Our analysis zooms in on a quite specific group of T2DM, which actually is not representative for diabetic inpatients, as we discussed in the manuscript (e.g. *Line 250-258*). As a fact, our result of DKD incidence is more similar to that of a national study carried out in representative T2DM outpatients in China (mostly are of urban population) [6]. With a mean duration of 8.1 years and similar diagnosing criteria of nephropathy, the incidence of diabetic nephropathy in that study was 14.4%. 4. Compared to DKD, DR in this analysis is more affected by the retrospective nature of our study and the way data were collected. Unlike DKD with its index (UACR, e GFR) for diagnosing all retrievable from the electronic laboratory records, results of ophthalmoscope examination were not necessarily recorded into the electronic records but rather were kept in handwritten records by the ophthalmologists, especially those without going through fundus fluorescein angiography. This increases chance of missing patients with their DR at early stages. So we must admit that our study is not for analyzing prevalence of diabetic complications, neither should the results taken as a typical incidence in hospital-based population. We mentioned this in the manuscript without extensively discussing at length. We could if you would like.  ***16. Line 177: A total of 164 T2DM patients were included in this study. In table 2, the authors reported 43 patients were treated with OAD, 121 patients were treated with insulin. The total number was 164. In line 177, the authors said that “only 1 patient was treated with diet only in SMH”. Please double-check the data “only 1 patient (0.6%)” whether they are right or not.***

Checked and corrected as ‘None was treated with diet-only on discharge in patients at SMH’ (*Line201-202*). It’s another error with negligence that shouldn’t have happened.  ***17. Line 179-181: In table 2, we did not find a significant difference in OADs between two hospitals (P=0.34). So please rephrase this sentence.***

Exactly. I have rephrased the expression in the description of RESULTS as well as in the DISCUSSION.  ***18. Line 251: You wrote about less patients in our study had initiated insulin treatment-however, it does not become clear less than what/where? Do you refer to the previously figures in the other study? If so, please give numbers for this statement as well.***

We compared insulin treatment on admission (previous treatment patients accepted as outpatients) in our patients with a former study carried out in T2DM outpatients. The ratio of ‘insulin-only’ and ‘orally anti-diabetic agents +insulin’ were 17.5% and 18.2%, respectively, in that study (6), which are nearly twice more frequent than ours. Thanks to your reminding, we have discussed more on this (*Line 265-270*).  ***19. Line288: Similar to my comment 18. Please add more information.***

Edited. As the addition of insulin to treatment has been most frequently verified as effective adjustment for patients with inadequate glucose control [3, 11], the recommended ‘basal-and-bolus’ or ‘basal plus’ insulin treatment for inpatient hyperglycemia management [12, 13] are not necessarily prime for post-discharge treatment. Instead, basal insulin is the commonest choice [11]. While in those with significant hyperglycemia who are unable to or unwilling to cope with complexity of Basal-and-bolus regimen, transition to premixed insulin treatment may be a better choice [14]. Besides, of note is the characteristic tendency to more profound post-prandial hyperglycemia in Chinese T2DM [15] which may also favors premixed regimen of insulin treatment. As you suggested, we’ve supplemented the manuscript with more information in *Line 306-309*, as well as in the following discussion in the manuscript.

***20. Line 324-330: Although patients in rural area has the NCMS, they can’t afford the financial burden because such new type of medications are not covered by NCMS. But in LHLH, only 57.5% of all subjects were covered by insurance, why these people were prescribed with more GLP-1A? Please discuss.***

Edited (*Line344-355*). The use of GLP-1RA as a newer class of antidiabetics has been getting more favorable evidences in its non-inferiority in glucose-lowering potency and extra benefits in less hypoglycemia and weight gain [3]. ADA guideline recommend its combination with basal insulin as a comparable choice to basal-and-bolus insulin treatment [16]. Much of the obstacle to the application of GLP-1RA might be economical consideration since it’s more costly than insulin regimen. Your concern of the relatively low coverage of insurance in our patients is surely reasonable. The reason why more patients were able to follow this new treatment could be multifaceted. For one thing, socioeconomic background as well as better education drive patient to choose advanced treatment and they are more willing to receive information from specialists to keep in pace with development. For another, more urban citizens don’t settle for basic medical insurance and they are more willing to invest in personal health insurance plans than the rural population. Hospital information system doesn’t record these personal insurance plans since patients get reimbursed by the insurance company after their discharge. This fact might also contribute to their readiness to receive more expensive treatment like GLP-1RA.

***21. Line333-340: Please add some references.***

Advice taken. While basal and bolus insulin regimen has been considered as mainstream of inpatient diabetes care, it requires self-management capabilities and good compliance of the patients post-discharge to make the best of it. We’ve added references concerning why basal-bolus insulin treatment could turn out to be a burden some patients unable to or unwilling to deal with. ***22. I can’t find table 5 was discussed or provide any useful information in the manuscript, if this is true, I recommend to delete this table.***

As we explained in your eleventh comment about the statistical methods, we’ve provided with necessary explanatory information and presented the results in Table5 (*Line247-248*) and discussed the inference of the analysis in DISCUSSION as well (*Line 377-381)*.

***23. Line353: In terms of generalizability, this study only included T2DM inpatients in two specific hospitals, the findings could be valid in similar populations and settings. Inpatients have higher blood glucose or sever conditions. A population-based study should be conducted, which might provide a different picture of the issue. Please add this in limitations section.***
Truly so. While discharge planning for transition remaining to be deemphasized is a general problem, our study is carried out in one particular clinical population in one administrative region. The results are limited in its scope and generalizability. Future studies are imperative to shed more lights on the problem. Thank you again for your understanding and insights. We’ve stated this in the limitations (*Line397-401*).

References

1.	Weng J, Ji L, Jia W, Lu J, Zhou Z, Zou D, et al. Standards of care for type 2 diabetes in China. Diabetes Metab Res Rev. 2016;32(5):442-58.

2.	American Diabetes Association. 15. Diabetes Care in the Hospital: Standards of Medical Care in Diabetes—2020. Diabetes Care, 2020, 43(Supplement 1): S193-S202.

3. Umpierrez G E, Reyes D, Smiley D, et al. Hospital discharge algorithm based on admission HbA1c for the management of patients with type 2 diabetes. Diabetes Care, 2014, 37(11): 2934-2939.

4.	Kong AP, So WY, Szeto CC, Chan NN, Luk A, Ma RC, et al. Assessment of glomerular filtration rate in addition to albuminuria is important in managing type II diabetes. Kidney Int. 2006;69(2):383-7.

5.	Leung G M, Lam K S. Diabetic complications and their implications on health care in Asia. Hong Kong Med J. 2000 Mar;6(1):61-8.

6.	Ji L, Hu D, Pan C, Weng J, Huo Y, Ma C, et al. Primacy of the 3B approach to control risk factors for cardiovascular disease in type 2 diabetes patients. Am J Med. 2013;126(10):925 e11-22.

7.	Gheith O, Farouk N, Nampoory N, et al. Diabetic kidney disease: world wide difference of prevalence and risk factors. J Nephropharmacol, 2016, 5(1): 49.

8.	Adler AI, Stevens RJ, Manley SE, Bilous RW, Cull CA, Holman RR, et al. Development and progression of nephropathy in type 2 diabetes: the United Kingdom Prospective Diabetes Study (UKPDS 64). Kidney Int.2003;63(1):225-32.

9.	Kramer H J, Nguyen Q D, Curhan G, et al. Renal insufficiency in the absence of albuminuria and retinopathy among adults with type 2 diabetes mellitus. Jama, 2003, 289(24): 3273-3277.

10.	Retnakaran R, Cull CA, Thorne KI, Adler AI, Holman RR, Group US. Risk factors for renal dysfunction in type 2 diabetes: U.K. Prospective Diabetes Study 74. Diabetes. 2006;55(6):1832-9.

11.	Perez A, Reales P, Barahona MJ, Romero MG, Minambres I, Group HS. Efficacy and feasibility of basal-bolus insulin regimens and a discharge-strategy in hospitalised patients with type 2 diabetes--the HOSMIDIA study. Int J Clin Pract. 2014;68(10):1264-71.

12.	Umpierrez GE, Hellman R, Korytkowski MT, Kosiborod M, Maynard GA, Montori VM, et al. Management of hyperglycemia in hospitalized patients in non-critical care setting: an endocrine society clinical practice guideline. J Clin Endocrinol Metab. 2012;97(1):16-38.

13.	American Diabetes A. 14. Diabetes Care in the Hospital: Standards of Medical Care in Diabetes-2018. Diabetes Care. 2018;41(Suppl 1):S144-S51.

14.	Wu T, Betty B, Downie M, Khanolkar M, Kilov G, Orr-Walker B, et al. Practical Guidance on the Use of Premix Insulin Analogs in Initiating, Intensifying, or Switching Insulin Regimens in Type 2 Diabetes. Diabetes Ther. 2015;6(3):273-87.

15.	Qian L, Xu L, Wang X, Fu X, Gu Y, Lin F, et al. Early insulin secretion failure leads to diabetes in Chinese subjects with impaired glucose regulation. Diabetes Metab Res Rev. 2009;25(2):144-9.

16. American Diabetes Association. 9. Pharmacologic Approaches to Glycemic Treatment: Standards of Medical Care in Diabetes—2020. Diabetes Care, 2020, 43(Supplement 1): S98-S110.
